# Video feedback compared to treatment as usual in families with parent–child interactions problems: a randomized controlled trial

**DOI:** 10.1186/s13034-015-0036-9

**Published:** 2015-02-12

**Authors:** Magnhild Singstad Høivik, Stian Lydersen, May Britt Drugli, Ragnhild Onsøien, Marit Bergum Hansen, Turid Suzanne Berg- Nielsen

**Affiliations:** Regional Centre for Child and Youth Mental Health and Child Welfare - Central Norway, Faculty of Medicine, The Norwegian University of Science and Technology, N-7491 Trondheim, Norway; St Olavs Hospital, Trondheim University Hospital, Division of Psychiatry, Trondheim, Norway; National Network for Infant Mental Health, Oslo, Norway; The Center for Child and Adolescent Mental Health, Eastern and Southern Norway, Oslo, Norway

**Keywords:** RCT, Intervention, Video feedback, Parent, Child

## Abstract

**Background:**

For the first time to our knowledge, short- and long-term effects of a multi-site randomized-controlled trial (RCT) of video feedback of infant–parent interaction (VIPI) intervention in naturalistic settings are published. The intervention targets families with children younger than 2 years old and parent–child interactions problems. Outcome variables were 1) observed parent–child interactions and 2) parent-reported child social and emotional development. Between-group differences of the moderating effects of parental symptoms of depression, personality disorders traits, and demographic variables were investigated.

**Method:**

The study had a parallel-group, consecutively randomized, single-blinded design; participants were recruited by health- and social workers. Seventy-five families received VIPI, and 57 families received treatment as usual (TAU). Videotapes of each parent–child interactions were obtained before treatment, right after treatment, and at a 6-month follow-up and coded according to Biringen’s Emotional Availability Scales. Parental symptoms of depression and personality disorder traits were included as possible moderators.

**Results:**

Evidence of a short-term effect of VIPI treatment on parent–child interactions was established, especially among depressed parents and parents with problematic interactions–and, to some extent, among parents with dependent and paranoid personality disorder traits. A long-term positive effect of VIPI compared with TAU on child social/emotional development was also evident. In a secondary analysis, VIPI had a direct positive effect on the depressive symptoms of parents compared with TAU.

**Conclusion:**

The findings of the study support the use of VIPI as an intervention in families with interaction difficulties.

**Trial registration:**

Current Controlled Trials ISRCTN99793905.

## Background

Based on the overwhelming evidence of the parent–child relationship being fundamental to child health and development, a number of prevention and treatment strategies targeting early dyadic difficulties have emerged. Three theoretical directions dominate the therapeutic work with parents and their young children: the representational [1-7], the interactional/behavioural [8-11], and methods integrating both of these theoretical views [12-14]. All of the theoretical approaches have implemented the use of video; however, interventions with a behavioural perspective more frequently. Video feedback has also been included in broader, intensive family treatment programs [13,15-17] and in more narrowly directed home-based interventions [18,19].

This study will focus on a video feedback parenting intervention developed by Maria Aarts: the Marte Meo method [20]. It is a home-based intervention considered to exist between the interactional/behavioral approaches and the representational approaches, and it has been used in work with troubled families since the 1980s by more than 10,000 therapists worldwide [21]. However, evidence from randomized-controlled trial (RCT) studies of this frequently used method is non-existent. The current trial will attempt to fill this knowledge gap by measuring the effect of a manual intervention based on Marte Meo elements: the video feedback of infant–parent interaction, or VIPI [22].

### Previous research on video feedback interventions

The use of video feedback was first introduced into work with families in The Netherlands [19,23] to help parents watch themselves from the “outside” [24-26]. Later, in addition to focusing on parental skills and behaviour, video feedback was used in more comprehensive psychotherapeutic work to enhance parental mentalization capacities [7,27,28]. Adding video to conventional treatment programmes has been shown to increase the treatment effect on parental sensitivity [29]. There are contradictory opinions regarding whether parents should be offered a widely focused treatment [30] or a treatment that targets sensitivity only, contending that “less is more” [29].

In representational therapies, therapeutic exchanges target parental representations of close relationships that prevail in the face of treatment, both in relation to the therapist and in the parents’ interactions with the child. When a video camera is introduced into the therapeutic setting, the video replay offers a more distant perspective of the parent–child relationship. In a triangulating space formed with the therapist, the parents are given the opportunity both for self-observation and to see the child as a separate human being, with a mind of its own [7,31].

In the interactional/behavioural approaches, behavioural transactions are thought to be the main source of change in the parent–child relationship on an implicit, unconscious level; that is, the child’s experience of being with the parents is modified through changed parental behaviours [8]. In these methods, the main components are the non-authoritarian stance of the therapist and the therapeutic goals selected by the parents, who are assisted in the positive reinforcement of existing competences. The Dutch video feedback interventions to promote positive parenting (VIPP) programs [14] are either behavioural (VIPP)/VIPP-sensitive discipline) or use a combined behavioural/representational approach (VIPP with a representational focus). The Ulm Model [32], the interactive guidance (IG) [33], video interactive guidance (VIG) [34], and video home training (VHT) [35], on the other hand, are mainly behaviourally oriented.

Although there are more studies on the effects of behaviour-oriented interventions than that of representational therapies [36], both methods have the same impact on parental behaviours, attitudes, and self-esteems, as well as on infants’ sleeping habits [5,27,36]. Video intervention therapy (VIT) [37] and the “watch, wait and wonder” method (WWW) [27,38] extract useful elements from both representational and behavioural views. The same applies to therapy using clinically assisted video feedback exposure sessions (CAVES), which was developed to change traumatized mothers’ relationships with their babies [28].

Two meta-analyses of parent–child interaction interventions revealed that short-term treatment directed at parental sensitivity was most effective [36,39]. However, since the meta-analytic findings were based on post-treatment evaluations without a follow-up measure, the effect over time remains uncertain [29,36].

For child outcomes, small to average effects on child behaviour were found in one meta-analysis [36]. Others have published findings of long-term positive effects on child flexibility and optimal ego–control in adopted girls, as well as decreased internalizing problems among both boys and girls [14,40].

Since the latest meta-analysis was published in 2008 [36], findings from new RCT studies have supported the existing evidence for the effectiveness of video feedback in comparison to controls, in improving parental sensitivity [41,42], the broader concept of parent–child interactions [43], or children’s externalizing and internalizing problems in maltreating families [41].

To our knowledge, there are only seven studies, four of which have an RCT design [5,44-46], that have examined the long-term effects of video feedback on parental sensitivity and child outcomes in full-term infants [5,32,40,44-47]. Of these studies, only two actually found effects on maternal sensitivity in mothers six months or more after intervention [5,46]. Yet, additional studies are necessary to establish knowledge regarding the long-term effects of video feedback interventions on both parent–child interactions and child outcomes [48]. In consonance with this, in addition to examining the short-term effects that VIPI might have on parent–child interactions, this study will focus on longitudinal effects (at a 6-month follow-up). The interaction will be measured using Biringen’s Emotional Availability Scales [49]. Emotional availability refers to caregivers’ affective attunement to their children’s needs and goals and involves the acceptance of a wide range of emotions, as well as the children’s emotional and behavioural response towards their parents [50]. Biringen uses the concept of sensitivity to denote a variety of parental qualities that keep adults warm and emotionally connected to their children, including responsiveness, an accurate perception of the children’s communication and an ability to smoothly resolve conflicts. The appropriateness and authenticity of the adult’s affect is, however, considered to be the single most important parental characteristic.

### Marte Meo guidance

In Norway and in other parts of Scandinavia, Marte Meo is the most widely implemented parenting intervention for families at risk during the first years after child-birth. In Norway, the method has primarily been used to treat parent–child interactional problems in community health and welfare services, in kindergartens, in work with adoptive parents and in child and adolescent psychiatry departments [20,51]. There exist three qualitative studies on the positive effect of the Marte Meo intervention on maternal sensitivity towards infants and on decreased maternal symptoms of depression [52-54]. Likewise, Marte Meo has been demonstrated to be useful as a means of supporting adoptive parents [55] and has shown a promising effect in a systematic, school-based intervention among slightly older children with externalizing behaviours [56]. A positive effect of a method related to Marte Meo, The Orion Project (Video Home Training), has also been published [19]. Maria Aarts and Harry Bieman developed this home visitation model to work with families with interaction problems [20]. Later, Aarts further developed the Marte Meo approach in accordance with the emerging “empowerment tradition” within social work [57] to enhance clients’ self-efficacy in dealing with their parental roles. The Marte Meo intervention comprises videotaping of parent–child interactions during daily activities. Only one element of their interactional capacities is focused on at a time, giving the parents the opportunity to move forward “step by step”.

### Moderators of effect

Among the parental factors that could possibly influence treatment, depression should be considered, as it is the psychiatric illness that most frequently occurs in the first year after birth and is known to negatively influence both parent–child interactions and child outcomes [58,59]. The prevalence of post-natal depression ranges from 8% to 15% internationally [60-62] and from 8.9% to 16.5% in Norway [63-66]. Video feedback has been implemented in treatment programs for post-natally depressed mothers and their infants [67]. Yet, so far, no effect modification of maternal depressive symptoms on treatment with video feedback has been reported [42,46]. Less information exists on parental personality disorders and how they affect interactional problems [68-72]. How parental personality disorders may serve as moderators of the treatment effects of video feedback is, to our knowledge, unexplored. If not severe, these conditions are often not addressed and might, therefore, be under-diagnosed in community settings. Consequently, self-report measures of symptoms of depression and personality disorders were included as possible moderators in this study.

Two child factors—child age and child gender—were included as possible moderators in the current inquiry because they have been proven to moderate the treatment effect in other video interventions with more positive effects observed in families with girls and older children [29,40,41].

Poverty, first-time or single parenthood, young age of parents, marital conflict, and lack of social support are considered to be pertinent factors in the ecological milieu that influences a child’s development [30,73]. Therefore, the moderating effects of these factors on intervention efforts are also of interest and will be examined in this inquiry.

### The current inquiry

Prior to the enrolment of participants in the study, the VIPI manual was developed to meet the requirements of a standardized intervention. The manual was developed for children up to 24 months of age; hence, the study sample was recruited accordingly. The manual uses the core elements of the Maria Aarts method, and offers a structural frame for the existing Marte Meo video intervention practice, with some principle differences. The only divergent points are the mandatory order of thematic sequences during the intervention, the limited (six to eight) number of meetings and the obligatory written homework between sessions (which were optional in the original practice).

### Aims

#### Main hypotheses

This RCT investigated, in a heterogenic community sample of families with interactional problems, whether VIPI would be more effective than standard care (TAU) received in the community.

Our first hypothesis was that parents receiving VIPI would benefit more from the intervention than parent receiving TAU. Hypothesized effects were: (a) increased parent–child emotional availability and (b) positive social and emotional development of the child compared with the TAU group. We also expected the differences in treatment effects to persist at the six-month follow-up.

#### Hypotheses of moderation

Second, we investigated whether parental depressive symptoms would influence our treatment effects. Our hypothesis was that depressive symptoms would not moderate the effect on parent–child emotional availability.

Furthermore, we explored the influence of personality traits on the effect of VIPI intervention on parent–child emotional availability. Our hypothesis was that parental personality disorder traits would negatively interfere with the treatment effect.

Finally, the moderating effects of different background variables on the treatment effect were investigated. We hypothesized that background variables, such as a family’s socio-economic status, experienced support from a network, and ongoing conflicts would influence the effect of VIPI on emotional availability, with positive effects occurring in families with high socio-economic status, high levels of experienced support and low levels of conflict. With regard to parental age and the parity of the attending child, we hypothesized that younger, first-time mothers would show a stronger effect of VIPI treatment. Child age and gender were also expected to be important; we hypothesized older children and girls to experience better outcomes from VIPI intervention.

## Methods

This was a naturalistic longitudinal multi-site RCT in urban and rural samples in Norway. It had a parallel-group, consecutively randomized single-blinded design.

### Study sample

**Figure 1:**
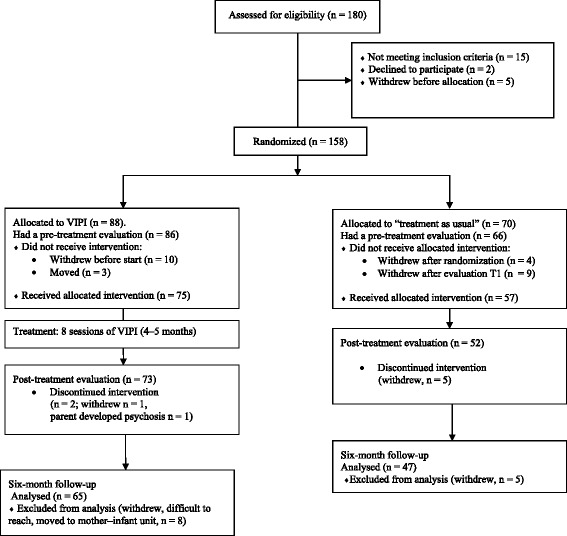
**Inclusion, randomization, and attrition in the study.**

From March 2008 to September 2012, 158 families were invited to attend the study by primary health and social workers in the cities of Trondheim and Oslo and in six rural communities in the eastern part of Norway (Table 1, Figure 1). Inclusion criteria were parent–child interaction problems and children aged 0 to 24 months at the time of inclusion. Interactional problems were widely defined by either the parents themselves or the recruiting health- or social workers. Since numerous recruiters from various community services participated in this naturalistic study, it has been difficult to estimate how representative our sample was in comparison to all families with interaction difficulties or how frequently interaction difficulties occurred in the population from which we recruited. Parents with ongoing psychosis, developmental disorders or substance abuse and parents with insufficient proficiency to fill out the questionnaires were excluded. The study had no child exclusion criteria because the professionals involved in the study considered that video feedback of parenting could be useful regardless of child characteristics. Only two fathers attended the study. In 23 families, both parents took part in the intervention; however only one of the parents was included in the study. In most families, the mothers chose to participate. Sixty-four per cent of the mothers (compared to 10.3% of the fathers) had parental leave at inclusion time; hence, mothers chose to participate largely due to practical reasons.

**Table 1:** **Sample characteristics**

**Characteristic**	**n or** ***mean*** **(sd)**	**%**
**Child characteristics**		
Child living with	140	
Living with both parents		82.9
Living with biological mother		15.7
Living with mother and stepfather		0.7
Living alternately with mother and father		0.7
Age at inclusion (months)	141 *7.3* (5.1)	
Child’s gender	141	
Boy		49.0
Girl		51.0
Cohabitant siblings	137	
First born child		72.0
Older siblings		28.0
**Parental characteristics**		
Gender participating parent	157	
Mothers		98.7
Fathers		1.3
Age of mothers at inclusion	140 *29.7* (5.6)	
Ethnic origin of mothers	96	
Norwegian		82.6
Other European		6.5
African		3.3
Asian		5.4
South American		2.2
Maternal educational level at inclusion	140	
Junior high school		5.7
Senior high school		12.1
Vocational education (1–2 years)		19.3
Bachelor degree		25.0
Master degree or higher		37.9
Ongoing education, mothers	130	
Yes		18.7
No		81.3
Age of fathers at inclusion	134 *32.8* (7.0)	
Ethnic origin of fathers	93	
Norwegian		89.8
Other European		6.8
African		2.3
North American		1.1
Fathers’ educational level at inclusion	135	
Junior high school		5.3
Senior high school		17.3
Vocational education (1–2 years)		19.5
Bachelor’s degree		30.8
Master’s degree or higher		27.1
Ongoing education, fathers	132	
Yes		13.3
No		86.7
Earlier/ongoing psychiatric illness	143	
Mothers		17.5
Fathers		5.6
Other partner		0.7
Family income, after tax (in 1000 NKr)	135 *33.9* (17.5)	
Experienced support	140	
Satisfied (very/a little)		90.0–99.3
Unsatisfied (very/a little)		0.7–10.0
Conflicts in close relations (partner, family, friends, colleagues)	127	
Never/hardly ever		62.6–87.1
Sometimes		4.4–29.4
Often/very often		4.0–11.4

Among the 152 families that had a pre-treatment evaluation, the parents reported problems in 50.9% of the cases; in the rest of the families, participation in the study was recommended by a health or social worker (49.1%). The health and social workers who recruited the families to the study reported maternal depressive symptoms (60–70%), worries about the child’s development (about 10%), insensitive parenting (about 10%), and interest in learning more about parenting (10–20%) as the most important reasons for recruitment to the study. However, participating parents reported differently about the reasons for participation: regulation problems (32.6%), parent–child interactional problems (14.5%), interest (10.8%), parental psychiatric disorders (3.6%), developmental delay (3.2%), worries about social development (2.4%) and a need for support (2.2%) were given as the main motives to attend the study. For 30.7% of the participants, the reasons were not reported, perhaps because these families were recommended to participate by health or social workers. Five families had contact with a child welfare service; one family had help economically, and four received “other support”.

### Procedure

Three trained research assistants with bachelor’s degrees in preschool education, nursing or social work visited the families in their homes. During the visit, parents completed the questionnaires and were videotaped while interacting with their children for 30 minutes in a natural everyday situation such as feeding, playing or nappy changing. These videotapes were later assessed according to a standardized observation measure, which was our main effect outcome. Evaluations with this observation measure were conducted for all included families at pre-treatment (baseline) (T1); post-treatment (2–3 months after baseline) (T2); and 6 months after the treatment had ended (T3). The study period lasted from 9 to 13 months (mean 11.5 months). After the T1 evaluation, the families were consecutively randomized to either a treatment group (VIPI) or a control group (TAU) in a 1–2–1–2 allocation ratio within each urban district or rural municipality by a clinical psychologist, who also served as a coordinator for those professionals in the communities who enrolled participants in the study.

All research assistants were blinded to the randomization status of the families in the work through assessment and data handling. A total of 152 videotapes of the parent–child interactions at T1, 125 at T2 and 112 at T3 were coded and included in the analysis. Four tapes were missing, and two tapes were damaged and could not be coded.

Self-report questionnaires addressing parental depressive symptoms and the assessment of the social and emotional development of their children were filled out at all three time points, whereas information about personality disorder traits was obtained at T1 (Table 2).

**Table 2:** **Descriptive statistics of EAS, BDI, DIP-Q, and ASQ:SE**

	**VIPI**			**TAU**		
	**n**	**mean**	**sd**	**n**	**mean**	**sd**
EAS score T1	86	137.10	28.75	66	139.19	27.73
EAS score T2	73	151.90	19.60	52	145.84	29.23
EAS score T3	63	153.40	22.33	47	156.15	19.25
BDI score T1	67	11.37	8.83	51	12.84	8.45
BDI score T2	63	9.17	7.42	42	9.55	7.50
BDI score T3	45	8.20	6.93	31	9.71	7.48
DIP-Q T1						
Cluster A	59	3.46	3.52	44	3.34	3.06
Cluster B	59	5.37	3.50	44	5.59	4.26
Cluster C	55	7.87	7.78	45	8.00	4.29
Paranoid	62	1.31	1.68	47	1.36	1.47
Schizoid	63	0.73	1.02	47	0.72	0.97
Schizotypal	65	1.29	1.47	45	1.31	1.66
Borderline	61	2.69	2.11	45	2.38	2.30
Histrionic	61	1.20	1.18	48	1.29	1.27
Narcissistic	63	0.83	0.93	48	1.04	1.17
Antisocial	65	0.83	0.76	48	0.81	1.07
Avoidant	61	1.96	2.05	47	2.13	1.87
Dependent	61	1.76	2.01	50	1.92	1.87
Obsess. comp	62	3.83	1.70	47	4.17	1.74
ASQ: SE score T1	35	33.86	23.23	25	26.66	15.73
ASQ: SE score T2	37	26.21	19.61	27	25.74	17.02
ASQ: SE score T3	26	20.44	13.45	27	25.00	16.53

EAS: Emotional Availability Scales.

BDI: Beck Depression Inventory.

DIP-Q: DSM IV and ICD-10 Personality Questionnaire.

ASQ:SE: Ages & Stages Questionnaires: Social Emotional.

Of the eight VIPI therapists, one had completed high school and seven had bachelor’s degrees in social work (two), nursing (two), physiotherapy, preschool education or child welfare education. All were certificated and experienced Marte Meo-therapists. Before the families were recruited to the study, the therapists were educated in the use of the VIPI manual during three 2-days training sessions and were supervised on one or more families by a licensed supervisor. During this supervision, the parents’ interactions with their children as well as the therapists’ feedback to the parents (both captured on videotapes) were discussed.

To ensure treatment fidelity of the therapists to the VIPI manual, videotapes of the therapists’ feedback to the parents during their interventions with their fourth VIPI families were checked by an experienced, licensed supervisor. Families in the VIPI group received eight video feedback sessions, with the last two sessions being tailored to meet individual family needs regarding any of the six topics in the manual. If both parents were included in the intervention, separate video tapes were obtained and individual feedback was given to each parent. Naturally, VIPI parents were also free to visit other health professionals for routine care. The TAU parents only received routine care at the well-baby units, but they were also free to seek help from others. Prior to the study, however, interveners of TAU were clearly informed that they could not give any form of video based feedback to the TAU families, and they were reminded of this during the study. VIPI interveners were also reminded not to “leak” information about the intervention to TAU interveners.

Nurses at the well-baby unit offered visits to all families in both groups at 4 and 6 weeks after delivery, and then at 3, 6, 8, 10, 12, 15, 18 and 24 months. The families also met with a physician from the well-baby unit when their children were 6 weeks, 6, 12, and 24 months old. Of the VIPI parents, 40.5% had visits with their health centre nurses (mean frequency 4.27). The families also received help from: psychologists (13.3%; mean frequency 2.42), physicians (20.0%; mean frequency 1.78), general practitioners (30.8%; mean frequency 1.07), specialists at somatic hospitals (2.5%; mean frequency 0.09) and “others” (1.8%; mean frequency 0.08). Of the TAU parents, 36.7% were followed by their nurses in the well-baby units (mean frequency 3.59), other health professionals as psychologists (5.9%; mean frequency 0.12), physicians (11.4%; mean frequency 0.92), specialists at somatic hospitals (1.8%; mean frequency 0.15), general practitioners (23.5%; mean frequency 0.75), or “others” (3.0%; mean frequency 0.50).

Socio-economic and demographic data were obtained at the time of inclusion in the study (Table 1).

### The VIPI manual

The Norwegian VIPI manual was developed by three experienced Marte Meo supervisors [22]. The manual describes guidance through several steps or levels for families with children under 2 years of age.

The method especially targets parental sensitivity and structuring, in relation to concerns addressed by the parents. At least six consultations are provided, with the opportunity for extra sessions related to any of the topics, if necessary. Both the videotaping and the feedback take place in the families’ homes. Weekly interventions are recommended, with a maximum intervention length of 3 months. Before each session, the therapist carefully selects 5–6 minutes of videotaped interactions between the caregiver and his or her child to enlighten one of the thematic elements from the manual. The video clips are then used in feedback sessions with the parents. For instance, in the first session, representative scenes of the child’s initiatives of contact with the caregiver are selected from two videotapes obtained in structured and non-structured contexts (e.g., during feeding and playing). Good parental practice is supported by a reflective dialogue between parent and therapist. Some of the sessions might be repeated; the speed of the progression depends on how the parents respond to the intervention. The families receive homework between sessions related to the newly addressed topics; for instance, parents are asked to register moments with experienced dialogue and turn-taking in their interactions with their infants.

The VIPI consists of six subsequent sessions which focus on these elements:

#### Initiative of the infants to contact caregivers and initiate pauses in the dyadic exchange

Addresses the infants’ initiatives to contact parents and their need for pauses in the dyadic exchange. For older children, this addresses children’s initiative to gain joint attention with their caregivers directed towards objects.

#### Responses of caregivers

Topics and issues that need to be worked are identified based on the mutual observations of the responses of parents and the timing of their responses to the contact initiatives of their infants/children. Adequate parental acknowledgement, support and affective responses are focused on.

#### Following the child

The main goal of this session is to encourage parents to support initiatives coming from their children. Following parental acknowledgement of their children’s initiative to contact them, parents are encouraged to wait until the children responds to ensure synchronous turn-taking and mutual exchange.

#### Naming

Parents are encouraged to articulate what is happening in the interactions by naming initiatives, intentions, emotions, relational activities, actions, and transitional situations.

#### Step-by-step guidance

In this session, the parental capacity to structure the interaction is addressed. The adults take the lead in a balanced way to help their children during and between tasks and activities.

#### Directing attention towards social interaction and exploration

In the last session, the therapist encourages parents’ support for their children’s exploration of their surroundings and for the expansion of joint focus (e.g., directing the child’s attention towards other people through comments, interpretations, songs or stories.

### Instruments

*Emotional Availability Scales (EAS)* [49]: a research-based way of understanding the quality of communication and connection between a parent and child. The EAS are based on attachment theory, as well as the theoretical work of Robert Emde [74]. The parent’s supportive attitude regarding the child’s explorations of its surroundings, while representing both a physically “secure base” and a receptive presence for the child’s emotional signals, is observed, as is the child’s contribution to the relationship. The actual dyad is videotaped and evaluated. The method has been validated [75-79] and consists of six dimensions assessing the bidirectional emotional availability between the child and the adult: 1) adult sensitivity, 2) adult structuring, 3) adult non-intrusiveness, 4) adult non-hostility, 5) child responsiveness, and 6) child involvement of the adult. Each topic contains seven features, each assessed on either a 3- or a 7-point scale representing the accurately observed capacity of both adult and child. The range of minimum to maximum scores is 42 to 174 points. High scores indicate good emotional availability in the dyad.

Because of the naturalistic, non-stressful context, 30-minute interactional sequences were videotaped. The videotapes were scored by four coders who were trained and certificated by Zeynep Biringen in the fourth edition of the EAS. The assessors’ educational backgrounds included either bachelor’s degrees in preschool education or specializations in clinical psychology or child and youth/adult psychiatry, and one of the coders was a postgraduate student in clinical psychology. All raters were blind to the randomization. Cronbach’s alpha was 0.97 at all three time points. Intra-class correlations were used to analyse the inter-rater agreement. In the mixed-effect model, the total variance adjusted for time point is the sum of three variance components: variance between individuals, variance between raters, and residual variance. It follows [80], (pages 437–441) that the between-rater, within individual intra-class correlation estimate is

$$ ICC=\frac{139.284}{139.284+22.973+139.739}=0.461. $$

The average Pearson correlation between the raters was 0.63. Averaging all 36 paired ratings resulted in practically the same Pearson correlation coefficient (results not shown).

*Beck Depression Inventory (BDI–II)* [81]: a self-report containing 21 issues. Each issue has four statements with increasing severity corresponding to the most accurate description of the situation over the last 2 weeks. The statements are scored from 0 to 3, where 0 indicates no specific problems, and 3 represents the most severe condition. The maximum score is 63, indicating major depressive symptoms. The interpretation of the scoring is as follows: 0–13: no indication for depression; 14–19: mild depressive symptoms; 20–28: moderate depressive symptoms; 29–63: severe depressive symptoms.

The scale is thoroughly validated in the research and is widely used in clinical practice [82,83]. Cronbach’s alphas ranged between 0.86 and 0.88 in this study.

*DSM IV and ICD-10 Personality Questionnaire (DIP-Q*) [84]: a 140 item true/false self-report scale addressing personality traits developed through the comparison of self-reported symptoms and diagnostic interviews. The scale addresses symptoms that meet diagnostic criteria for 10 personality disorders according to DSM IV, 8 according to ICD-10. Only the DSM IV related items (102 statements) were used in the current study. The DIP-Q was validated in the Swedish population in 1998 [85]. The overall sensitivity of the scale in the Swedish study was 0.84, its specificity was 0.77, and its agreement with the DSM cluster was found to be acceptable (Cohen’s kappa 0.45–0.63). Self-report vs interview correlations of dimensional scores for each personality disorder clusters were high: ICC = 0.60–0.78.

The DIP-Q has been used in other Scandinavian studies [23,71,84]. Cronbach’s alpha in the current investigation was 0.77.

*The Ages & Stages Questionnaires: Social Emotional (ASQ:SE)* [86]: a screening tool to identify children who might be at risk for social and emotional difficulties. It comprises a series of eight questionnaires that correspond to age intervals; in our study, we have used the schemas for 6, 12, 18, 24, 30, and 36-month-old children. The questionnaires address seven behavioural areas in the child’s development: self-regulation, compliance, communication, adaptive functioning, autonomy, affect, and interaction with people. The questions are adapted to normal developmental milestones for each age span with a positive expectation of behaviours. However, some of the questions are reversed. The questions are answered by “Yes”, “Sometimes”, or “Not yet”, corresponding to point values of 0, 5, or 10 points. Low scores give no indication of delayed social and emotional development, high scores give indication for further investigation.

The validity of the ASQ:SE has been established through a standardized assessment performed by experienced raters and has shown an overall agreement of diagnostic classification of 93% (81% to 95%), with a sensitivity of 78% and specificity of 95% [86].

### Statistics

Prior to the study, a power analysis was executed, based on an earlier reported effect size [36]. In this study, a Cohen’s d of short-duration video feedback family treatment at 0.68 was reported. With an expected standardized difference between the VIPI and TAU groups of 0.5, 60 families were needed in each group to give a power of 78% at a 5% significance level.

The intervention effect was investigated by an analysis of covariance, ANCOVA [87]. We investigated whether the effect of our intervention was mediated through either emotional availability (Step 1) or child social/emotional development (Step 2). Putative moderators of the VIPI’s effect on the outcome variables were also examined (Step 3).

*Step 1*: Regression analyses were performed with the total EAS score [75] at T2 and T3, respectively, as dependent variables, and with the EAS score at T1, the treatment group and their products (i.e., Intervention group × EAS score) as covariates.

*Step 2*: To investigate the treatment effect on the social/emotional development of the children, we also performed ANCOVAs with ASQ:SE at T2/T3 as dependent variables. Treatment group, ASQ:SE at T1 and their products (i.e. Intervention group × ASQ:SE) were covariates. Because we had to compare scores from different ASQ:SE forms due to the wide range in the ages among the children at each time point, we chose to use adjusted ASQ:SE scores to allow for the varied contents and cut-off values of the different forms. Our ASQ:SE variables were calculated from age-adjusted means in a no-risk population, as given by the results published in the ASQ:SE manual (Table A9, page 89) [86].

*Step 3:* The moderating effects of depressive symptoms, personality traits, and background data of the parents on the treatment effect found in previous analyses in step 1 were investigated by including the actual variable and its product with the treatment group as covariates. For child social and emotional development, only the moderating effect of parental depressive symptoms was investigated.

The inter-rater reliability of our observational measure—the EAS—was analysed as follows: 36 individuals were drawn at random, 12 from each of the three time points. Each was rated by two raters, drawn from a pool of four raters. All six combinations of raters rated two individuals at each of the three time points. To calculate the inter-rater correlation coefficient (ICC), we used a mixed-effect model with time point (1, 2, 3) as categorical covariate (also known as a fixed factor) and with individual and rater as crossed random factors. With this analysis, we could determine whether certain raters tended to give consistently higher scores than other raters.

In addition, we calculated Pearson’s correlation coefficient for each six pairs of raters, where each pair had rated six combinations of individuals and time points, and then averaged these six coefficients.

A total of 5.6% of the values of the DIP-Q scales were missing. Moreover, 3.96% of the BDI values at T1, 2.62% of the BDI values at T2, and 0.54% of the BDI values at T3 were missing; however, only 69 parents had completed the BDI total scores at all three time points, 96 had completed BDI total scores at T1 and T2, and 71 had done so at T1 and T3. For the various ASQ:SE forms, 0 to 10.3% of the values were missing. Due to the small percentages of missing values, we chose to exclude cases with missing values rather than employ imputation.

A two-sided p < 0.05 was chosen to indicate statistical significance. Ninety-five percent confidence intervals (CI) were reported where relevant. The ICC was calculated using Stata 12. All other analyses were conducted using SPSS 19.

### Ethics

The Regional Committee for Research Ethics in Mid-Norway approved the study, with reference number 1.2007.2176. All participants gave written informed consent to participate. Our study is registered in the International Standard Randomized Controlled Trial Number register, with reference number ISRCTN99793905.

In two families, the parenting was considered harmful for the child, and Child Welfare Services were notified.

## Results

**Figure 2:**
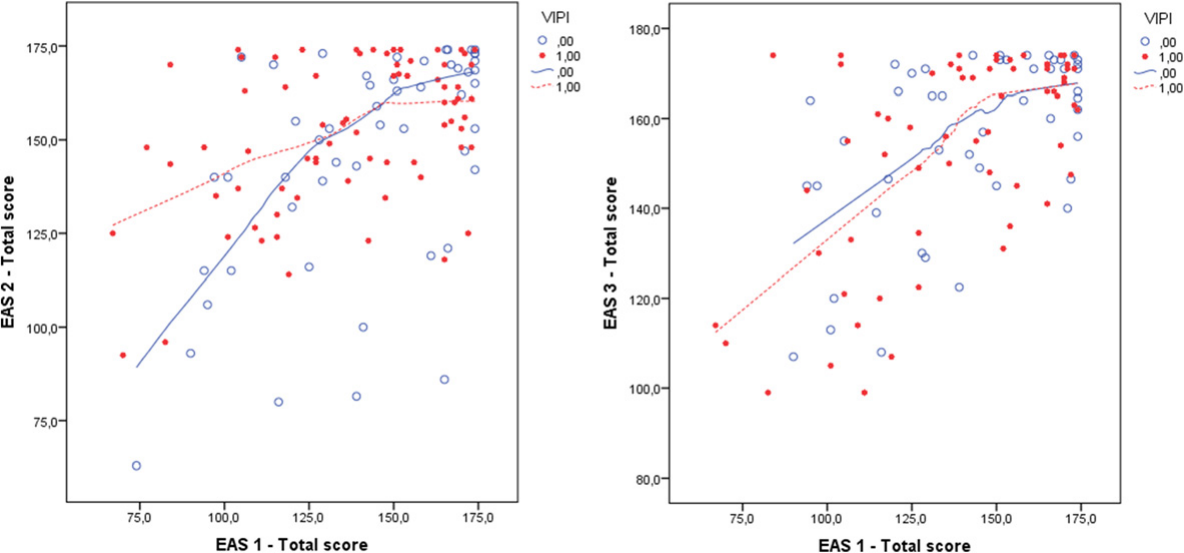
**EAS total scores at T1 compared with T2/T3 in VIPI vs TAU groups.** EAS 1/EAS 2/EAS 3 total score: the Emotional Availability Scales score at inclusion (T1), after treatment (T2) and at the 6-month follow-up (T3). VIPI = 1(0) for the treatment group (TAU).

In Step 1 of the analysis, the VIPI treatment group improved their parent–child emotional availability after treatment (T2) with a total EAS score 8.5 points higher than the controls who received TAU (95% CI 0.81 to 16.20, p = 0.03). However, the effect depended on the EAS scores at baseline; the lower the emotional availability in the parent–child dyad in the VIPI group, the greater the intervention effect that was found compared with that of the TAU group (Intervention group × EAS score: p = 0.04) (Table 3, Figure 2). We therefore chose to keep this effect-modifying variable in our further analyses. Consequently, the effect of VIPI increased substantially, to 47.3 points, compared with TAU (95% CI 8.78 to 85.78, p = 0.02).

**Table 3:** **Effect of VIPI (differences between VIPI and TAU) on EAS score at T2 adjusted for EAS score and not adjusted/adjusted for BDI at baseline: regression coefficient estimate, CI, and p-value for VIPI at different values of EAS score and BDI score at baseline**

**EAS score at T1/ Sample percentile**	**Not adjusted for BDI**	**BDI = 5**	**BDI = 15**	**BDI = 25**
	**B value/95% CI/p**	**B value/95% CI/p**	**B value/95% CI/p**	**B value/95% CI/p**
EAS score = 97	**20.49 (6.57 to 34.41)**	10.96 (−13.98 to 25.91)	**25.13 (11.45 to 38.81)**	**39.30 (21.62 to 56.97)**
(10th percentile)	**0.004**	0.15	**<0.001**	**<0.001**
EAS score = 116.5	**15.10 (5.15 to 25.05)**	**15.79 (4.59 to 26.98)**	**19.14 (9.12 to 29.16)**	**33.31 (18.24 to 48.38)**
(25th percentile)	**0.003**	**0.006**	**<0.001**	**<0.001**
EAS score = 143	**7.79 (0.15 to 15.41)**	−3.16 (−12.91 to 6.58)	**11.00 (3.12 to 18.89)**	**25.17 (11.36 to 38.98)**
(50th percentile)	**0.05**	0.52	**0.007**	**< 0.001**
EAS score = 165	1.70 (−8.38 to 11.78)	−9.92 (−21.38 to 1.53)	4.25 (−5.76 to 14.25)	**18.42 (3.24 to 33.59)**
(75th percentile)	0.74	0.089	0.40	**0.018**
EAS score = 172	0.23 (−11.64 to 11.18)	−12.07 (−24.56 to 6.29)	2.10 (−9.10 to 13.29)	**16.27 (0.26 to 32.27)**
(90 percentile)	0.97	0.058	0.71	**0.046**

The regression equation:

EAS 2 = 69.24 + 0.650 EAS1 + 33.114 VIPI – 0.302 EAS1 × VIPI – 1.382 BDI + 1.355 BDI × VIPI.

Treatment group: VIPI = 1(0) for treatment group (TAU).

EAS: Emotional Availability Scales, BDI: Beck Depression Inventory.

High EAS scores indicate good emotional availability in the parent–child dyad. BDI = 5 indicates no parental depressive symptoms; BDI = 15 indicates mild depressive symptoms and BDI = 25 indicates moderate depressive symptoms.

Bold numbers: significant differences in the level of ≤ 0.05.

Because the EAS minimum score is 42, not 0, we used centered EAS scores in the following analyses for easier interpretation of our further outputs. Since the effect of VIPI (i.e., the differences between the groups) is a function of the baseline EAS, percentiles of EAS were chosen to illustrate it. For families showing low emotional availability in their interactions at T1 (EAS total scores between 97 and 116.5 points, representing the 10th and 25th percentiles in our material), a highly significant positive change in favour of the treatment group was found (see Table 3, column “Not adjusted for BDI”). For families with middling EAS scores at T1 (EAS total score 143, representing the 50th percentile), the increase was less, but significant. Within the well-functioning dyads, with total EAS scores between 165 (75th percentile) and 172 (90th percentile) points, no significant difference between the VIPI and TAU groups was found.

**Figure 3:**
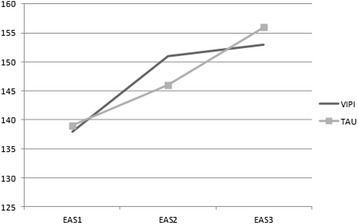
**Mean EAS scores at baseline, after treatment and at the 6-month follow-up.**

At the 6-month follow-up (T3), both the VIPI and TAU groups exhibited higher emotional availability in their parent–child interactions with an increased mean total EAS scores compared with T1 (Table 2, Figure 3). For the VIPI group, 90.8% of this increase was seen during the intervention period; for the TAU group, the corresponding increase was only 39.1%. However, there were no significant differences in the total EAS scores between groups, either for the families with low emotional availability at T1 or when a possible moderating effect of parental depressive symptoms was included in the analysis (Table 4, Figure 2).

**Table 4:** **Effect of VIPI (differences between VIPI and TAU) on EAS score at the 6-month follow-up; T3, adjusted for EAS score and BDI at baseline: Regression coefficient estimate, CI, and p-value for VIPI at different values of EAs score and BDI score at baseline**

**EAS score at T1/Sample percentile**	**BDI = 5**	**BDI = 15**	**BDI = 25**
	**B value/95% CI p**	**B value/95% CI p**	**B value/95% CI p**
EAS score = 97	−7.21 (−21.44 to 7.02)	−3.94 (−17.50 to 9.62)	6.55 (−13.23 to 26.33)
(10th percentile)	0.32	0.57	0.51
EAS score = 116.5	−6.11 (−17.02 to 4.79)	0.77 (−9.74 to 11.27)	7.65 (−9.30 to 24.60)
(25th percentile)	0.27	0.89	0.37
EAS score = 143	−4.62 (−13.78 to 4.54)	2.26 (−5.59 to 10.11)	9.14 (−5.85 to 24.13)
(50th percentile)	0.32	0.57	0.23
EAS score = 165	1.50 (−7.60 to 10.60)	3.50 (−5.98 to 12.97)	10.38 (−5.17 to 25.92)
(75th percentile)	0.74	0.47	0.19
EAS score = 172	−2.99 (−15.21 to9.23)	3.89 (−6.69 to 14.46)	10.77 (−5.35 to 26.89)
(90th percentile)	0.63	0.47	0.19

The regression equation:

EAS 3 = 100.943 + 0.437 EAS1 – 16.103 VIPI + 0.056 EAS1 × VIPI – 0.630 BDI + 0.688 BDI × VIPI.

VIPI = 1(0) for the treatment group (TAU).

EAS: Emotional Availability Scales, BDI: Beck Depression Inventory. High EAS scores indicate good emotional availability in the parent–child dyad. BDI = 5 indicates no depression; BDI = 15 indicate mild depressive symptoms and BDI = 25 indicates moderate depressive symptoms.

**Figure 4:**
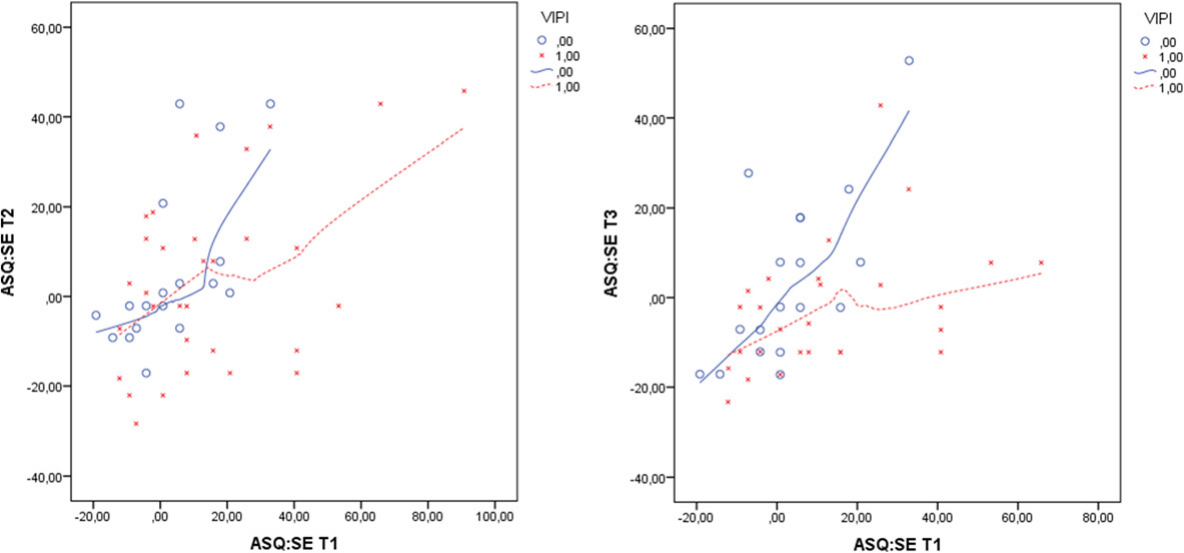
**Between-group effects of VIPI/TAU on ASQ:SE scores at T2/T3 compared with T1 scores.** VIPI = 1(0) for the treatment group (TAU). Centralized ASQ:SE values (using American norms for mean values) were applied for easier interpretation of the different ASQ:SE forms at each time point.

In Step 2, we investigated the between-group effect of VIPI on the child’s capacity for self-regulation, compliance, adaptive functioning, autonomy, affect, and interaction with others using ASQ:SE. At T2, no significant differences were found between the VIPI group and the TAU group (see Table 5). At T3, however, in the VIPI group, we found significantly less parental concern regarding delayed social and emotional development in the children (Table 5, Figure 4). This result persisted when parental depressive symptoms at T1 were controlled for; therefore, the treatment effect was not merely the result of an improvement in parents’ depressive symptoms. There was no significant moderating effect of maternal depressive symptoms at T1 on the VIPI effect on child development measured with ASQ:SE (p = 0.44).

**Table 5:** **Effects of VIPI (differences between VIPI and TAU) on ASQ:SE/BDI after intervention (T2) and at the 6-month follow-up (T3) adjusted for ASQ:SE/BDI at baseline: B-values, confidence-intervals and p-values**

	**ASQ:SE T2**	**ASQ:SE T3**	**BDI T2**	**BDI T3**
	**B-values, 95% CI p**	**B-values, 95% CI p**	**B-values, 95% CI p**	**B-values, 95% CI p**
ASQ:SE T1	−7.22 (−17.74 to – 3.33)	**−13.79 (−25.27 to – 2.31)**		
	0.17	**0.02**		
BDI T1 = 15			−0.91 (−3.44 to 1.62)	**−2.64 (−5.24 to −0.04)**
			0.48	**0.047**
BDI T1 = 25			−3.53 (−7.94 to 0.88)	**−6.52 (−11.01 to −2.03)**
			0.12	**0.005**

BDI = 15 indicate mild depressive symptoms reported by the Beck Depression Inventory; BDI = 25 indicates moderate depressive symptoms.

Bold numbers: significant differences in the level of ≤ 0.05.

In Step 3, parents with few depressive symptoms (BDI total score of 5) and low emotional availability in interactions with their children had no significant effect of VIPI (Table 3). Interestingly, when the mothers had ongoing mild-to-moderate depressive symptoms (BDI total score of 15 and 25 points), there was an expected increase in the EAS score after treatment in the VIPI group compared to the TAU group (Table 3). Because only four parents had severe depressive symptoms, we chose to omit higher BDI scores from the analysis.

The results indicated that the more severe the depressive symptoms of the parents, and the more problematic the initial emotional availability between parents and children, the better the treatment effect of VIPI. For high-functioning families with fairly good or good emotional availability (EAS scores between the 75th and 90th percentiles), the picture was more complex: co-occurring moderate depressive symptoms among parents (BDI total score of 25 points) increased the effect in favour of the VIPI group. However, in cases of low BDI scores (5 points) and fairly good to good EAS scores, the results tended to favour the TAU group, with borderline significance at an EAS score of 172 points (Table 3).

For personality disorder traits, the effects on VIPI intervention were more complex. Contrary to what we hypothesized, we found no modifying effects on intervention effect of Clusters A, B, or C, or of schizotypal, schizoid, borderline, histrionic, antisocial, avoidant, or obsessive-compulsive personality disorder traits (Table 6). For dependent personality disorder (DPD) traits, there was a highly significant effect of VIPI in families with the lowest interactional competence and with DPD scores at cut-off level for diagnosis (five traits confirmed). Within families with good interactional competence, only three parents had DPD scores at the cut-off or higher; therefore, no analysis was possible (Table 6). Also paranoid personality disorder (PPD) traits were associated with more positive intervention effects; the lower the interactional competence and the higher PPD scores, the better the effect of VIPI (Table 6). We found no significant effects between VIPI and TAU for different EAS scores at T1 when the interaction between PPD and VIPI was taken in to the ANCOVA analysis with a PPD score of 0 (Table 6). Because we had few participants with scores over the cut-off value (five traits confirmed), we investigated the moderator effect of two and four PPD traits. Within families with low EAS scores, we found a significant between-group effect in favour of VIPI when the PPD score was 4. For families with higher emotional availability, we had inadequate data to perform the analysis. For narcissistic personality disorder (NPD) traits, there was a better VIPI effect with no NPD traits in families with low EAS scores. For the more well-functioning dyads with EAS scores between 165 and 172 points, we found that even two narcissistic traits could deteriorate the effect of VIPI (Table 6). Few in our sample had high NPD scores.

**Table 6:** **Results from ANCOVA regression equations (differences between VIPI and TAU) with EAS score at T2 as dependent variable, VIPI, different values of EAS at T1 and different personality disorder Clusters scores and personality scores as covariates**

**EAS score T1/Percentile**	**97 (10th)**	**116.5 (25th)**	**143 (50th)**	**165 (75th)**	**172 (90th)**
DIP-Q item					
Cluster A	ns	ns	ns	ns	ns
Cluster B	ns	ns	ns	ns	ns
Cluster C	ns	ns	ns	ns	ns
Paranoid					
Paranoid_0	15.56_a_	9.23_a_	0.64_a_	−6.50_a_	no data
Paranoid_2	–	–	**11.22**_**a**_ *	4.22_a_	no data
Paranoid_4	**37.06**_**a**_ ***	**30.85**_**a**_ ***	no data	no data	no data
Schizoid					
Schizoid_0	ns	ns	ns	ns	ns
Cut-off (4)	ns	ns	ns	ns	ns
Schizotypal					
Schizotypal_0	ns	ns	ns	ns	ns
Cut-off (5)	ns	ns	ns	ns	ns
Borderline					
Borderline_0	ns	ns	ns	ns	ns
Cut-off (5)	ns	ns	ns	ns	ns
Histrionic					
Histrionic_0	ns	ns	ns	ns	ns
Cut-off (5)	ns	ns	ns	ns	ns
Narcissistic					
Narciss_0	**35.10**_b_***	**27.41**_**b**_ ***	**16.97**_**b**_ **	8.30_b_	5.90_b_
Narciss_2	11.59_b_	3.90_b_	−6.54_b_	−**15.21**_**b**_ *	−**19.10**_**b**_ *
Antisocial					
Antisocial_0	ns	ns	ns	ns	ns
Antisocial_2	ns	ns	ns	ns	ns
Avoidant					
Avoidant_0	ns	ns	ns	ns	ns
Cut-off (4)	ns	ns	ns	ns	ns
Dependent					
Dependent_0	**17.63**_**c**_ *	9.10_c_	−2.49_c_	no data	no data
Dependent_3	–	–	**15.72**_**c**_ **	no data	no data
Cut-off (5)	**48.97**_**c**_ ***	**40.44**_**c**_ ***	no data	no data	no data
Obsessive compulsive					
Obs.comp._0	ns	ns	ns	ns	ns
Cut-off (4)	ns	ns	ns	ns	ns

In all analyses, (VIPI × EAS percentile score) is kept in the ANCOVA with p = 0.03–0.002. An abbreviation with _0, _2, and _4 means zero, two, or four traits in the respective personality disorder category, and the actual cut-off values for the respective personality disorder given in ( ).

VIPI = 1(0) for the treatment group (TAU). EAS: Emotional Availability Scales. DIP-Q: DSM IV and ICD-10 Personality Questionnaire.

_a_ = p (VIPI × Paranoid score) < 0.05.

_b_ = p (VIPI × Narcissistic score) < 0.01.

_c_ = p (VIPI × Dependent score) < 0.01.

*p < 0.05, **p < 0.01, ***p < 0.001.

ns = not significant.

**Figure 5:**
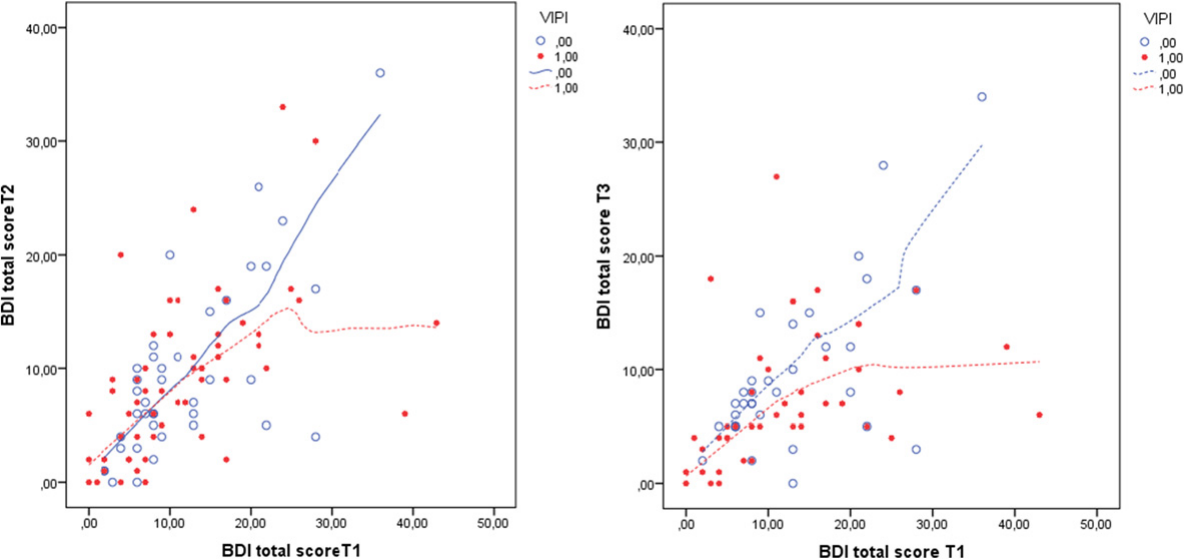
**Between-group effects of VIPI/TAU on BDI scores at T2/T3 compared with T1 scores.** VIPI = 1(0) for the treatment group (TAU).

In the step 3 analysis, we found a significant moderating effect of depressive symptoms on the VIPI effect; the more serious the depressive symptoms, the better the effect from VIPI compared with TAU. Thus, we performed secondary analyses to investigate whether VIPI treatment also affected the depressive scores of the parent compared with TAU as an outcome measure at T2 and T3. We found that, as the seriousness of parental depressive symptoms increased, the effect of VIPI on depressive symptoms became more positive. Moderate parental depressive symptoms at T1 (BDI total score of 25) predicted a highly significant drop in depressive symptoms measured at T3 in the VIPI group compared with the TAU group (Figure 5, Table 5). For milder depressive symptoms (BDI scores of 15), a small but significant difference between groups was found (Table 5). At T2, no significant VIPI effect on the BDI measure was found when depressive symptoms at 25 points were used as a covariate in the ANCOVA (Table 5).

For background variables such as cohabitant status, income, experiences of practical and emotional support, experience of conflict in close relations a number of older or younger siblings of the participating child (Table 1), no significant moderating effects were found (p = 0.08–0.94). Parental age or educational level, child gender or age did not significantly affect treatment outcomes (p = 0.13–0.99).

## Discussion

In this naturalistic randomized-controlled study, we examined the effect of a parenting intervention—VIPI—compared with TAU, on three outcome measures: observed parent–child emotional availability (EAS), child social-emotional development (ASQ:SE), and parental symptoms of depression (BDI). We examined a heterogeneous sample of families with interactional problems recruited from the health and social services. In addition, we investigated the putative moderating effects of parental depressive symptoms and personality disorder traits measured at baseline (T1) on parent–child emotional availability. Finally, the moderator effects of family socio-economic status, family support and conflict levels, child age and gender on parent–child emotional availability were examined. For the outcome measure of child social and emotional development, only the moderator-effect of parental depression was analyzed. Evidence for short-term, but no long-term, effects on emotional availability in parent–child interactions was found. Further, at 6-month follow-up, differences between the VIPI and TAU groups in children’s social-and emotional development and in parents’ depressive symptoms became evident. Parental depressive symptoms, paranoid, dependent and narcissistic personality disorders traits, as well as the initial EAS scores, moderated the effect on the EAS score at T2 between the VIPI and TAU groups. No moderating effects from the background variables or from symptoms of the other parental personality disorders were found. In following paragraphs, the various results will be discussed in detail.

### Differences between VIPI and TAU on emotional availability at post-treatment evaluation (T2)

Both groups of families, whether randomized to the VIPI intervention or TAU, improved their observed parent–child interactions, as measured by the EAS scores at T2. This improvement was largest in the VIPI families with the lowest initial emotional availability in the dyad compared with the controls, with significant differences between groups at the post-treatment evaluation. We have not found other RCT studies showing a positive effect of intervention for those parents with the most problematic parenting skills. Parents with less interactional competence often have less capacity for mental representations of their relationships with their children [88,89]. In this study, the video clips visualization of what was taking place in the interactions along with substantial support provided by the therapist, might have strengthened a meta-cognitive capacity or, possibly, an “observational self” in these parents.

VIPI helped families to break unhealthy interaction circles at an earlier stage. In a healthy parent–child relationship, according to Tronick’s Model of Mutual Regulation, the child needs to experience that negative affective states can be changed to positive states through a parental capacity for interactional adjustment and the repair of “mismatches” [90]. The child is believed to develop internal representations of social interactions with parents as positive and repairable, which is fundamental for the child’s development of self-efficacy and efficient coping strategies in the face of adversity. Moreover, neurobiological brain research supports the importance of improved emotional availability between parent and child during the most crucial period for the development of the right prefrontal cortex, an important area for affect and stress regulation and for executive functioning in the child [91].

Consistent with findings from meta-analysis [29], the differences in EAS scores between the VIPI and TAU groups just after treatment might be explained by the use of video, which increased treatment effects, not only on parental sensitivity, but also on parent–child interaction competence as a whole. It is commonly stated that “pictures don’t lie”; moreover, pictures evoke emotions in a different way than words do [52]. With guidance from the VIPI therapists, parents may be able to see for themselves possible mismatches between their children’s signals and their own responses. Further, through VIPI intervention, parents can become observers of their own increased competence, as therapists meticulously points out the beneficial sequences of parent–child interactions. This activity may both reassure parents and boost their self-esteem within their parental roles. Researchers have argued that for parents, the primary source of information about inherent parental competence is through feedback from interactions with their infants [92]. Consistent with a theory of change in addiction research, it is likely that self-observed change (in comparison to receiving the feedback in normal consultations, without video) is more motivating for the maintenance of change in parental behaviour [93]. Possible misinterpretations of what happens in the parent–child interactions might also be resolved when a VIPI therapist articulates his/her own perceptions of the child’s nonverbal cues.

The EAS include a measure of parental hostility observed in the interactions with the child; accordingly, an increased EAS score might also reflect a decreased level of hostility in the relationship. Negative parental emotions related to children or parenting are often not uncovered in usual care [94]. However, the use of video might help parents with negative feelings to gain necessary emotional distance when a situation is more difficult than joyful. Stress caused by negative interactions with a child (for instance, if the child has excessive crying spells) might reduce parent’s reflective functioning, capacity for self-regulation, and therefore capacity to recognize mental states in the child correctly and to stay calm and soothing [95].

As in many intervention studies, one cannot rule out that the early positive intervention effect seen in the VIPI group at T2 was merely due to the overall more intensive treatment exposure experienced by the VIPI families in comparison to the TAU families, since the VIPI group received the VIPI intervention in addition to TAU.

### Parents with symptoms of depression

Parental depressive symptoms served as an important moderator of the effect of VIPI compared with TAU through the whole range of EAS scores. Parents with more depressive symptoms, regardless of their initial interactional competence, appeared to benefit most from VIPI, exhibiting increased emotional availability in the parent–child dyad compared with TAU. To our knowledge, video feedback has not previously shown to have a specific beneficial effect on parent–child interactions among parents with depressive symptoms. Since two other effect studies on video intervention found no moderating effect of parental depression [42,46], the effect might be specific to VIPI. Researchers seem to agree that patients’ depressive symptoms are associated with deficits in episodic memory (both visual and verbal) [96]. Since depressed persons also have a tendency both to verbalize about themselves and to recall earlier episodes more negatively than non-depressed persons [97,98] the “empowerment” quality of VIPI might be particularly effective, given the intervention’s almost exclusively focus on positive parent–child interactional sequences. Further, a time limited, structured intervention that targets one issue at a time through the use of both verbal and visual input might be especially beneficial for individuals with impaired concentration, perseverance and executive functioning typical of depressive symptoms. Another advantage of video is its unique potential for studying children’s signals and parental behaviours at a micro-level; slow-speed replay and the opportunity to rewind might facilitate an increase in parental sensitivity.

Feelings of inadequacy and self-blame are frequently present among depressed parents who struggle in their interactions with their children, especially when a child’s temperament is experienced as difficult [52,99]. Parenting problems or having a child with poor interactional competence may also “mask” parental depression or even induce it [100,101], creating a situation that can be easily overlooked during short visits to the well-baby unit [102].

### Parents with minor interaction problems

The VIPI intervention did not have any effect on fairly well functioning parent–child dyads. This “ceiling effect” should be of no major concern, since well-functioning parent–child dyads seldom seek help. A similar effect was also found in a previous study with another video feedback method [18]. Thus, VIPI is hardly suitable as a broad preventive measure, especially because we found a tendency, even if just of borderline significance, for parents with good interactional competence and few depressive symptoms to have better outcomes with TAU. This reflects findings from a 2008 study [44], in which the children became less securely attached following video feedback intervention. In fact, competent parents might have their natural interactional flow disturbed if they become more self-conscious and struggle to become “even better”; for instance, by trying to be much more responsive and, thereby, becoming intrusive and leaving the child with less self-reliance [103,104]. “Good enough” parents should instead be reassured that they have inherent competence [105].

### Differences between VIPI and TAU on emotional availability at follow-up 6 months post-treatment (T3)

The promising VIPI effect compared with TAU seen after intervention (T2) was not replicated at the 6-month follow-up (T3) as presented in Figure 3. However, because both groups experienced considerable improvements in their emotional availability, we might expect that the TAU group had good support from the professionals in the health and welfare systems dealing with the variety of interactional problems within these families. It is also possible that the three home visits by the same supportive research assistants to all the families in both groups represented an intervention in itself. The female research assistants did the filming, had lengthy interviews with the parents, and assisted with completion of the questionnaires, activities that might have instigated increased parental reflection and awareness of their children.

Finally, a longer treatment period or the addition of booster sessions following ended intervention might have contributed to sustained improvement in the parent–child interactions. Egeland and his co-workers have argued that each stage of child development requires different parental skills to match the needs of the developing child [30]. Thus, booster sessions or a longer treatment period might ensure parental responsiveness as children grow older.

### Differences between VIPI and TAU on child social/emotional development

Our findings revealed a delayed, but substantial, effect of VIPI on the children’s capacities for self-regulation, compliance, adaptive functioning, autonomy, affect and interaction with others, measured with the total ASQ:SE score at the 6-month follow-up after the completion of treatment (T3). We know that an early capacity for emotion regulation may prevent developmental trajectories for both internalizing and externalizing problems [106-108] and may provide protection against later psychiatric disorders [109,110]. Because this “sleeping effect” was not reflected in a corresponding difference between groups in the EAS score at T3, mechanisms other than a direct link between the observed interactions and actual child development must be involved. Brazelton has argued in his Touchpoints model that child development occurs in bursts followed by regressions and pauses [111]. Because of such “bursts”, it could take some time before the increased emotional availability in the parent–child dyad will affect child development; hence, the differences in emotional availability between groups, which occur just after treatment (T2), first manifest in the child at T3. This finding corresponds to that of a study of video feedback intervention in autistic children, in which increased child social skills, not mediated by intervention effects on parenting, was found in the treatment group compared to controls [112]. Still, one cannot rule out that parental perception of child development evident in the parent-reported ASQ:SE improved more in the VIPI group not because the child had changed much, but because the parents had developed an increased tolerance for minor child problems because of the intervention. This assumption is supported by findings in another Scandinavian study where parents’ assessment of child development with the ASQ:SE was strongly predicted by maternal stress [113].

### Differences between VIPI and TAU on parental depressive symptoms

In a secondary analysis, in which we used parental depressive symptoms, not as a moderator but as an outcome, no differences between groups were found right after treatment, but highly significant differences between groups were seen at the 6-month follow-up in favour of the VIPI group (see Table 5). This finding might merely represent a “sleeping effect” of the increased emotional availability (EA) at T2 at which point the EA was estimated to be significantly higher in VIPI families than in TAU families (see Figure 3). Another scenario is a putative synergetic effect between the reduced concerns for the child’s development and the improved EA, which may have mitigated parental depression symptoms in the VIPI group. How to efficiently treat postpartum depression to achieve a long-term effect on parents’ mood, parent–child interactions, and children’s development has, thus far, been minimally explored [17]. Earlier research on interventions targeting only the depressed parents has shown that such methods might fail to bring about changes in the parent–child relationship or in child development [17,114,115].

VIPI intervention improved the depressive symptoms of parents throughout the study period, which lasted from 9 to 13 months. Among post-natally depressed women, several studies have indicated that without intervention, stability or recurrence of depressive symptoms might be expected in this period [116,117] with one study showing elevated maternal depressive scores at a 3-years follow-up [117].

### Differences between VIPI and TAU on background variables as moderators

We found that none of the background variables interfered with the VIPI effect. Our sample is difficult to compare with either the normal or the multi-risk samples presented in the Dutch meta-analyses [36,39] as SES variables in a sample drawn from a less homogeneous population than the Norwegian one could possibly affect treatment outcomes greatly.

### Differences between VIPI and TAU on PD traits as moderators

To our knowledge, the possible moderating role of deviant parental personality traits on parent–child interactions has not been previously examined; hence, we included such a measure in this study. Within the families with the most compromised parent–child interactions, we found that some parental personality disorder traits had an impact on the VIPI effect in both a positive and a negative direction compared with TAU. Presence of dependent and paranoid personality traits actually served as important moderators in favour of the VIPI intervention, whereas absence of narcissistic traits increased the effect of VIPI compared with TAU. From clinical experience, we know that dependent persons welcome new ideas from others because of their own feelings of inadequacy. However, the effect over time is unclear, because the lack of self-confidence and clinging behaviours are usually profound [118]. Being allocated to the TAU group might also be experienced as more stressful by dependent parents.

One might also suspect that the use of video clips represents a tangible, reassuring element for paranoid people. These individuals’ mistrust in the intentions and actions of others [119] is likely to negatively affect intervention. However, the transparency and clarity afforded by the use of video ensures that there is no hidden agenda, which might be relieving for someone with paranoid traits and help him/her gain trust in both the therapist and the intervention efforts.

For parents with fairly good emotional availability in interactions with their children, the presence of even very few narcissistic traits disfavoured VIPI intervention. The easily violated, slightly narcissistic parent in these high-functioning groups might be disturbed by the fact that they are not perceived as being as exceptional as they thought they were during VIPI intervention, which could possibly lead to a weakening of their interactional competence. Just as likely, when parents with narcissistic traits watch video clips of themselves, their self-preoccupation might increase, and they may become even less aware of their children.

### Methodological issues

The ICC on the EAS scores in our study was 0.461. In some contexts, this value would be considered low. However, it should be noted that this low ICC is caused by the relatively large residual variance (139.739). The inter-rater variance (22.973) is, by far, the smallest of these variance components, so the contribution to the total variance from inter-rater variance is practically negligible. Even if this variance component were 0 instead of 22.973, the ICC would still be 0.499.

The average Pearson correlation between the raters was 0.63. The Person correlation coefficient and the variance components from the mixed model address different issues. A Pearson correlation coefficient of 0.63 can be considered moderately high, but it does not tell the entire story. For example, if one rater consistently rated scores exactly 20 points higher than another rater, the Pearson correlation between the two would be 1.0. On the other hand, the relative magnitude of the inter-rater variance in the mixed model tells us that there are no large systematic differences between the raters.

As far as we know, there is no potential bias interfering with our analysis.

### Limitations

The history of the seriousness and duration of ongoing depressive symptoms and/or earlier depressive episodes and/or other affective disorders was not available for investigation; neither was information concerning ongoing medication. For parents in our inquiry with moderate depressive symptoms, intervention directed only at the parent–child interactions was effective in decreasing their depressive symptoms significantly. This might not be the case for the most seriously depressed parents, since such were not present in our sample. Therefore, a limitation of our inquiry is that we do not know whether parental cognitive problems related to the most serious depressive states might interfere with the intervention effect.

In 23 families, both parents took part in the VIPI intervention; in most families, the mothers chose to participate in the study. Consequently, no data of the non-participating parents was analysed.

Our follow-up period was limited to 6 months after treatment. A longer follow-up period could provide information about further progress in emotional availability in the parent–child interactions.

### Clinical implications

Our study indicates that VIPI intervention has an impact on emotional availability in parent–child relationships. Unexpectedly, a specific VIPI effect compared with TAU in families with depressed parents was also found, regardless of existing interactional competence, resulting in increased emotional availability in parent–child interactions. Our findings, together with the long-term main effects on depressive symptoms, support the use of VIPI interventions among depressed mothers with young children. Some clinicians might contend that it is best to treat parental depressive symptoms before working with parent–child interactions; however, our findings give no indications of the necessity of such a sequence.

Even if the study failed to prove long-term effects on emotional availability compared with TAU, the 6-month follow-up evaluation revealed effects on child development. In their clinical work, VIPI therapists experience that one or two “booster sessions” within 6 months of the end of the intervention can enhance the positive effect on the parent–child relationship. However, investigating the effect of such “booster sessions” was not within the scope of our study.

## Conclusion

A short-term effect of a parenting intervention, VIPI, was found in comparison with TAU in families with low emotional availability in parent–child interactions, especially among parents with symptoms of depression. Long-term evidence was also found for VIPI effects on parental depressive symptoms and on children’s social and emotional development. Furthermore, our findings give some indication for the use of VIPI intervention among parents with certain personality disorder traits.
